# Clinical remission of a critically ill COVID-19 patient treated by human umbilical cord mesenchymal stem cells: A case report

**DOI:** 10.1097/MD.0000000000021429

**Published:** 2020-07-31

**Authors:** Bing Liang, Junhui Chen, Tao Li, Haiying Wu, Wenjie Yang, Yanjiao Li, Jianchun Li, Congtao Yu, Fangang Nie, Zhaoxia Ma, Mingxi Yang, Mingying Xiao, Panrong Nie, Yanfeng Gao, Chuanyun Qian, Min Hu

**Affiliations:** a Department of Critical Care Medicine, Baoshan People's Hospital, Baoshan; b Intervention and Cell Therapy Center, Peking University Shenzhen Hospital, Shenzhen; c Yunnan Yasheng Medical Technology Co., Ltd.; d Emergency Department of the First Affiliated Hospital of Kunming Medical University, EICU/MICU; e Yunnan Key Laboratory for Basic Research on Bone and Joint Diseases & Yunnan Stem Cell Translational Research Center, Kunming University; f Yunnan Jici Institute for Regenerative Medicine Co., Ltd., Kunming; g Department of Infectious Diseases; h Department of Neonatology, Baoshan People's Hospital, Baoshan; i School of Life Sciences, Zhengzhou University, Zhengzhou, China; j Boten International Stem Cell Hospital, Boten, Laos.

**Keywords:** 2019 novel coronavirus, case report, COVID-19, critically ill, mesenchymal stem cell

## Abstract

**Rationale:**

The COVID-19 cases increased very fast in January and February 2020. The mortality among critically ill patients, especially the elder ones, is relatively high. Considering many patients died of severe inflammation response, it is urgent to develop effective therapeutic strategies for these patients. The human umbilical cord mesenchymal stem cells (hUCMSCs) have shown good capabilities to modulate the immune response and repair the injured tissue. Therefore, investigating the potential of hUCMSCs to the treatment of COVID-19 critically ill patients is necessary.

**Patient concerns:**

A 65-year-old woman felt fatigued and had a fever with body temperature of 38.2^°^C, coughed up white foaming sputum. After 1 day, she had chest tightness with SPO_2_ of 81%, and blood pressure of 160/91 mm Hg.

**Diagnose:**

According to the guideline for the diagnosis and treatment of 2019 novel coronavirus infected pneumonia (Trial 4th Edition), COVID-19 was diagnosed, based on the real-time RT-PCR test of SARS-CoV-2.

**Interventions:**

After regular treatment for 12 days, the inflammation symptom of the patient was still very severe and the potential side effects of corticosteroid were observed. Then, allogenic hUCMSCs were given 3 times (5 × 10^7^ cells each time) with a 3-day interval, together with thymosin α1 and antibiotics daily injection.

**Outcomes:**

After these treatments, most of the laboratory indexes and CT images showed remission of the inflammation symptom. The patient was subsequently transferred out of ICU, and the throat swabs test reported negative 4 days later.

**Lessons:**

These results indicated the clinical outcome and good tolerance of allogenic hUCMSCs transfer.

## 1 Introduction

In December 2019, the 2019 novel coronavirus (now called SARS-CoV-2) infected pneumonia (COVID-19) outbreak began. As of March 4, 2020, SARS-CoV-2 had infected 80,424 people (among whom 2984 were death cases) in China and 12,578 people (among whom 234 were death cases) in other 73 countries and regions.^[1]^ It was reported that the elder patients were likely to get more severe symptoms, and the ICU admission ratio of them was higher than that of the younger patients. Besides the ground glass opacity in the lung, another typical diagnosis characteristic of the critically ill patients was the decrease of lymphocytes along with the increase of neutrophils. The ICU admission patients have high levels of Interleukin-6 (IL-6), Granulocyte-Colony Stimulating Factor, IL-8, IFN-γ inducible protein 10 (IP-10), monocyte chemotactic protein 1, and TNF-α (tumor necrosis factor) in the serum, indicating the occurrence of cytokine storm.^[2,3]^ Persistence of cytokine storm will thus cause severe organ injury and death.^[4]^ Because that there are no standard treatments to overcome the cytokine storm up to now, these critically ill patients are always treated with glucocorticoid. But in most cases, the treatment of glucocorticoid will cause severe side effects including osteoporosis and immunosuppression, or even delay the clearance of the virus.^[2,5]^ Therefore, it is very urgent to develop novel strategies to treat these critically ill patients.^[6]^

Mesenchymal stem cells (MSCs) have been used to treat type 2 diabetes, autoimmune disease, spinal cord injury, graft-versus-host disease, and other diseases with very good safety.^[7,8]^ Among different kinds of MSCs, the umbilical cord mesenchymal stem cells (hUCMSCs) can be easy to obtain and be cultured. hUCMSC has shown immunomodulation and tissue repair effects with low immunogenicity, which makes it a very ideal candidate for allogenic adoptive transfer therapy. It was also suggested that hUCMSC had the potential to treat H5N1 infection induced acute lung injury, which showed similar inflammatory cytokine profile to that of COVID-19.^[9]^

Here, we reported a critically ill elder female patient in China infected with SARS-CoV-2. The characteristics of the vital signs, CT images, clinical laboratory profiles, and major immune cell changes were investigated. The clinical outcome and potential of hUCMSC adoptive transfer therapy were also discussed.

## 2 Case presentation

On January 27 (day 0), 2020, a 65-year-old woman felt fatigued and had a fever with body temperature of 38.2^°^C, coughed up white foaming sputum. Considering that she had flown from Wuhan on January 21, 2020, she was immediately sent to the nearby hospital, and the throat swabs were collected. Then, antibiotics and phlegm reducing drugs were given for supportive treatment. On day 1, she had chest tightness, with SPO_2_ of 81% and blood pressure of 160/91 mm Hg. On the same day, the real-time RT-PCR result showed SARS-CoV-2 positive, and the X-ray examination showed ground glass opacity in her right lung. Interferon α (IFN-α) inhalation treatment was performed. On day 2, she felt chest tightness and had more difficult breathing, along with shortness of breath in the morning. In the afternoon, she was admitted to the infectious disease department of the Baoshan People's Hospital (Grade-A Tertiary Hospital) for better treatment.

On day 2, the clinical laboratory examination showed that the white blood cell count was in normal range, but the neutrophil percentage increased to 87.9%, along with the lymphocyte percentage decreased to 9.8%. According to the guideline for the diagnosis and treatment of 2019 novel coronavirus infected pneumonia (Trial 4th Edition),^[10]^ the patient was treated with antiviral therapy of lopinavir/ritonavir and IFN-α inhalation, combined with moxifloxacin, Xuebijing, methylprednisolone, and immunoglobulin. To reduce hypoxia and prevent respiratory muscle fatigue of the patient, the noninvasive mechanical ventilator was used under the guidance of the hospital specialist group.

On day 3, the patient could breathe easily under the ventilator, with normal body temperature but paroxysmal cough. Considering that she got severe diarrhea from January 30 night to January 31 morning, electrolyte replacement and rehydration were given for supportive treatment. In order to lower the blood glucose level (postprandial glucose level around 9.6–14.6 mM), insulin was given subcutaneously. On day 4, the diarrhea symptom remitted, but the patient showed severe electrolyte disturbance. The white blood cell count increased to 12.16 × 10^9^/L, among which the neutrophil percentage increased to 92.4%. The C-reaction protein increased to 44.64 mg/L, along with erythrocyte sedimentation rate increased to 88 mm/h. Under the cooperation of multidiscipline team, the patient was diagnosed as critically ill type COVID-19 along with acute respiratory failure and acute diarrhea. Diabetes and hypertension remained to be further determined.

On day 5, the patient showed no diarrhea and no shortness of breath at rest, but showed paroxysmal cough with a small amount of white sputum. The white blood cell count continuously increased to 13.92 × 10^9^/L, among which the neutrophil percentage increased to 95.1% and lymphocyte decreased to 2.9%. From day 5 evening to day 6 morning, the patient began to breathe fast with a respiratory rate of 35 to 44/min, which could not be improved by adjusting the parameter of the ventilator. The blood oxygen saturation was continuously lower than 86% to 90%. Under the guidance of the COVID-19 specialist group, the patient was urgently transferred to the ICU, and invasive intratracheal intubation was performed to decrease the respiratory distress.

From day 6 to day 8, the white blood cell count slightly decreased, among which the percentage of neutrophil increased to 82.2% and the percentage of lymphocyte increased to 12.5% (both were still abnormal). In the early morning of day 8, the patient got a gastrorrhagia with the amount around 230 mL. Considering the low levels of red blood cell count and hemoglobin, she was diagnosed as anemia that might be caused by immune or inflammation-related hemolysis. To modulate the immune cell ratio, thymosin α1 was given from day 7. Although a blood transfusion was performed on day 8, the red blood cell count (2.76 × 10^12^/L) and hemoglobin concentration (92.00 g/L) were still very low on day 9. On day 10, the serum bilirubin continuously increased, with the concentrations of DBil to 43.8 μM and I-Bil to 29.5 μM, indicating that liver injury possibly happened. The concentrations of C-reactive protein (CRP) (82.69 mg/L), procalcitonin (0.102 ng/mL), D-dimer (4.76 μg/mL), and ProBNP (670.2 pg/mL) were very high. Although the white cell count was in normal range (8.38 × 10^9^/L), the neutrophil percentage began to increase again to a very high level (92.4%). All these results indicated that the effects of glucocorticoid, antiviral drugs, and antibiotics might not work very well, and the gastrorrhagia was suspected to be caused by the side effects of glucocorticoid.

On day 11, considering the severe organ injury caused by an inflammatory response and side effects, the glucocorticoid and antiviral therapy were withdrawn under the guidance of the specialist group. On day 12, the physical condition of the patient was reevaluated. It was confirmed that she was diagnosed as critically ill type COVID-19, with severe pneumonia (mixed type), acute respiratory distress, multiorgan injury (liver, respiratory system, and blood), moderate anemia, hypertension, type 2 diabetes, electrolyte disturbance, immunosuppression, acute gastrointestinal bleeding, and other symptoms. The informed consent was obtained by the family member and patient to perform hUCMSCs adoptive transfer therapy. The treatment scheme was then discussed and approved by the ethics committee of the hospital, and consent forms were signed by the family member before the treatment.

hUCMSC product was manufactured in an ISO 9001:2015 certified manufacturing facility using standard operating procedures under good manufacturing practice conditions according to the National Medical Products Administration Guidance for industry and additional requirements for manufacturing of human cells, tissues, and cellular and tissue-based products. The umbilical cord was obtained from full-term human placenta of a healthy donor by caesarean section. Fully informed consent was obtained several weeks prior to the delivery. hUCMSCs were isolated according to the procedures described by Seshareddy et al.^[11]^ Briefly, umbilical cord was washed with sterile 0.9% (w/v) saline solution (Cisen Pharmaceutical Co, Ltd (Shandong, China)) to remove the blood. Blood vessels were carefully removed after incising the cord lengthwise. Wharton's jelly was dissected into small fragments (1–2 mm^2^ pieces) and treated with 0.2% collagenase for 2.5 hours at 37°C and then treated with CTS TrypLE Select Enzyme (Gibco, USA) for 30 minutes at 37°C with agitation. hUCMSCs were seeded in 25 cm^2^ flasks (Corning, USA) and maintained in culture medium including minimum essential medium-α (Biological Industries, Israel) and 5% human platelet lysate (Mill Creek Life Sciences, Taiwan, China). On day 3, nonadherent cells were removed. When the confluence reached 70% to 80%, cells were passaged by being treated with CTS TrypLE Select Enzyme (Gibco, USA). At passage 3, hUCMSCs were characterized according to the International Society for Cellular Therapy Guidelines. For the hUCMSCs used here, they were expanded until passage 5 and then washed, harvested, and cryopreserved in 10% (v/v) dimethyl sulphoxide (Sigma-Aldrich, USA). The products were inspected and stored in a controlled, continuously monitored liquid nitrogen storage tank. Karyotyping/G-banding of cellular products was normal. Cellular identity from a single source was confirmed by short tandem repeats test. The cells showed spindle shape morphology when attached to plastic. hUCMSCs were macroscopic clumps free and without contamination by pathogenic microorganisms (bacteria, mycoplasma, syphilis, hepatitis B virus, hepatitis C virus, human immunodeficiency virus, cytomegalovirus, human parvovirus B19, Epstein-Barr virus, human papillomavirus, human herpesvirus 6, and fungi) or endotoxin (≤0.25 EU/mL). More than 95% of the hUCMSCs were positive for CD90, CD105, CD44, CD73, and less than 2% of them were positive for CD45, CD19, CD34, CD11b, and HLA-DR. The viability was more than 90%. Using osteogenic differentiation medium, chondrogenic differentiation medium, and adipogenic differentiation medium following the manufacturer's instruction (Biological Industries, Israel), these hUCMSCs showed the ability to differentiate into osteoblasts, chondrocytes, and adipocytes, respectively. Before administration, cryopreserved cells were thawed, washed to remove dimethyl sulphoxide, and resuspended in 0.9% saline solution supplemented with 5% human albumin (CSL Behring AG, Switzerland) at a final density of 5 × 10^5^ cells/mL. The percent viability of the hUCMSCs was determined by trypan blue exclusion before infusion. The viability was more than 90%. The hUCMSCs were released for transplantation when they fulfilled release criteria including the cell number, viability, safety (sterility, mycoplasma, and endotoxin), and identity (surface markers). The product was transported from the manufacturing facility to the hospital in a validated shipping system at a controlled temperature of 4°C to 8°C measured by a data logger, which was inspected and transported by a trained doctor.

**Figure 1:**
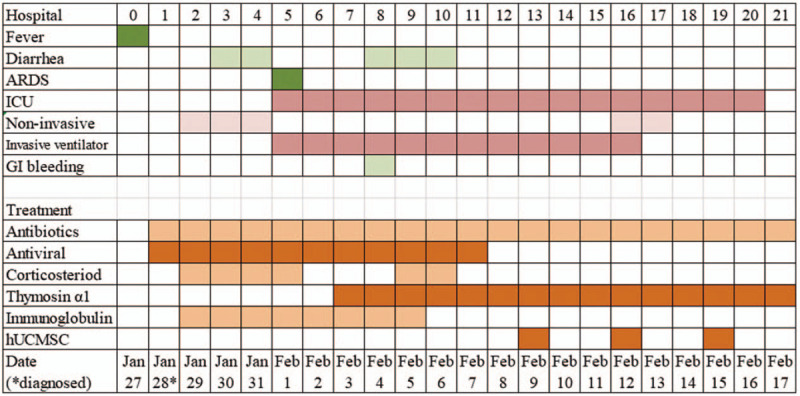
The major symptoms and treatment of the critically ill COVID-19 patient.

**Figure 2:**
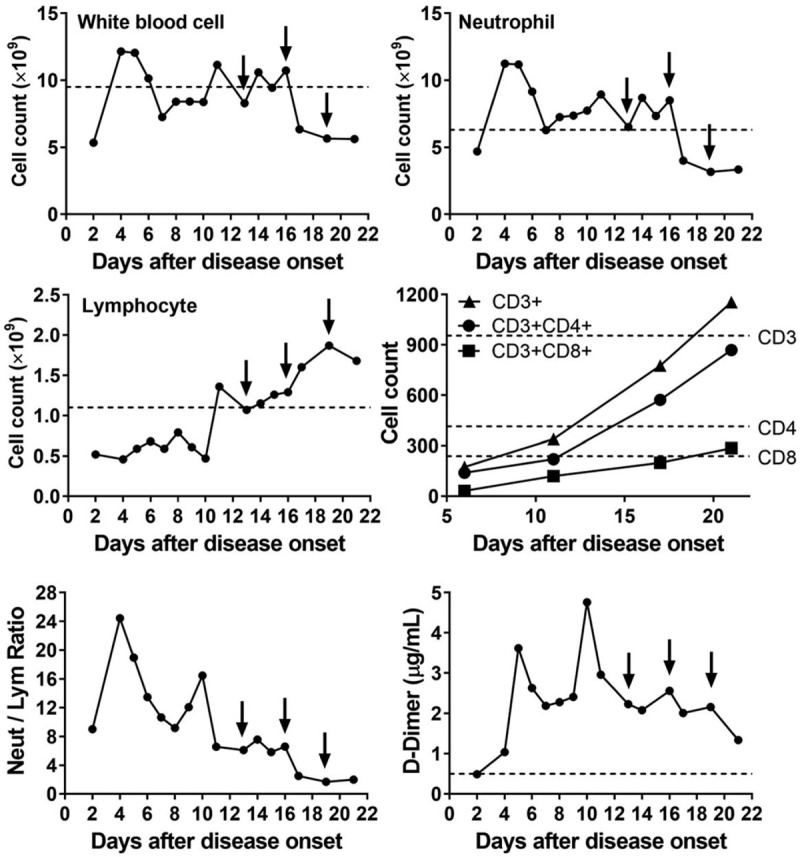
The dynamic changes of the immune cell counts of the patient. The arrows indicate the day of hUCMSCs therapy. To the white blood cell (normal range 3.5–9.5 × 10^9^/L), neutrophil (normal range 1.8–6.3 × 10^9^/L) and D-dimer (normal range 0–0.5 μg/mL), the dash line indicates upper threshold. While to the lymphocyte (normal range 1.1–3.2 × 10^9^/L) and T cell subsets, the dash line indicates lower threshold. hUCMSC = human umbilical cord mesenchymal stem cell.

As shown in Fig. 1, the allogenic hUCMSCs were administrated intravenously 3 times (5 × 10^7^ cells each time) on days 13, 16, and 19. During the treatment, antibiotics were given to prevent infection, and thymosin α1 was also given. After the first time of adoptive transfer, no obvious side effects were observed, indicating it was well tolerated. As shown in Table 1, after the second administration, the concentrations of serum bilirubin, CRP, ALT, and AST gradually reduced, along with some other vital signs also improved. The trachea cannula was then pulled off, and the patient could ambulate on the ground on day 17. As shown in Fig. 2, after the second administration, the white blood cell count and neutrophil count decreased to the normal level, along with the lymphocyte count increased to the normal level as well. More importantly, the counts of CD3^+^ T cell, CD4^+^ T cell, and CD8^+^ T cell also remarkably increased to normal levels. The neutrophil-to-lymphocyte ratio and D-dimer levels also decreased gradually. It was also suggested that the anti-inflammatory effects of thymosin α1 together with corticosteroid (from day 7 to day 12 in Fig. 2) might be not very effective, indicating that hUCMSCs might reduce the inflammation response, and help the recovery of antiviral T cell and injured tissues when combined with other immunomodulation agents. Considering the characteristics of hUCMSCs, we speculated that they might be homing to the injured tissues (especially to the lung), and then elicit anti-inflammatory function.

**Table 1:**
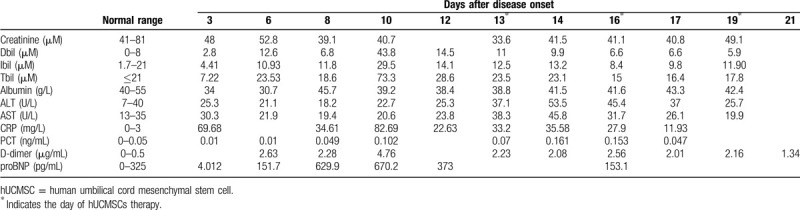
The major clinical laboratory characteristics of the patient.

**Figure 3:**
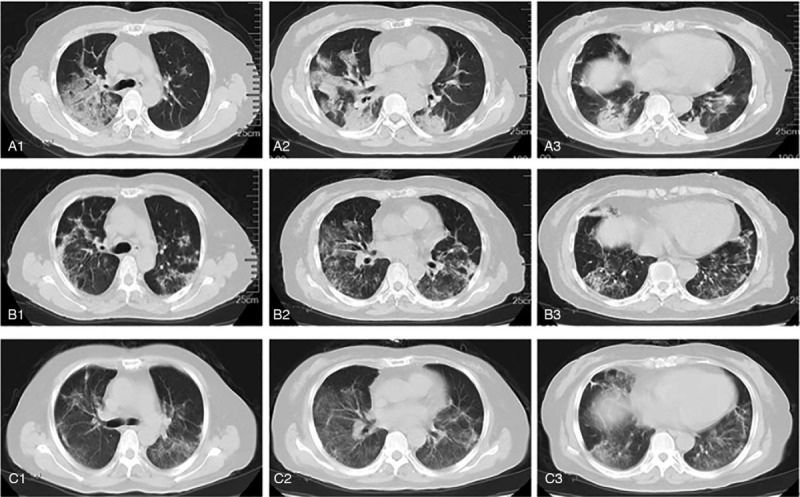
The typical CT images of the lung. A1–A3, CT images on day 2 indicate that there are lesions and mass density increasing shadow in both left and right lung. The ground-glass opacity, nonhomogeneous density, and air bronchus can be seen in the right lung. B1–B3, CT images on day 20 indicate the relief in both left and right lung. Only some Stripe shadow and small pieces of ground-glass opacity can be seen. C1–C3, CT images on day 25 indicate the further relief in both left and right lung. Most of the ground-glass opacity lightened, or even disappeared.

As shown in Fig. 3, by comparing the chest CT images taken on day 2 to day 20 and day 25, it can be seen that the pulmonary inflammatory reaction was greatly alleviated. On day 21, the patient was transferred out of ICU, and most of the vital signs and clinical laboratory indexes recovered to the normal level. The throat swabs tests reported negative on both day 21 and day 23. On day 30, the patient was discharged from hospital after recovery.

## 3 Discussion

Acute respiratory distress syndrome (ARDS) caused by inflammation and severe lung injury is the main cause of death in severe COVID-19 patients.^[4]^ Mesenchymal stem cells have good immunoregulation ability and can inhibit inflammatory response. Previous study proved that MSC is safe for ARDS treatment and has a protective effect on H5N1-associated acute lung injury. Therefore, MSC was considered to treat a critically ill COVID-19 patient here.^[12,13]^

After the administration of hUCMSCs, many clinical indexes and symptoms of the patient were improved. The counts of CD3^+^ T cell, CD4^+^ T cell, and CD8^+^ T cell remarkably increased to the normal level, indicating the reversal of lymphopenia, which is a common feature of the COVID-19 patients and associated with disease severity and mortality.^[4]^ Therefore, inhibition of inflammation and modulation of immune system by MSC are possibly effective on the treatment of COVID-19 patient.

Although the clinical benefits of hUCMSCs were indicated and tested in this case, the mechanism remained to be investigated by a well-designed clinical trial and more data collected. We proposed that the possible effects of hUCMSCs might be anti-inflammation and tissue repair to COVID-19 patients, and it was also reported that MSCs could down regulate proinflammatory cytokines and chemokines and increase IL-10 and VEGF which could promote the lung repair. But the detailed molecular pathways involved need to be revealed in the future.^[14]^ On the other hand, the dose and cell source of MSCs, and the combination strategy, diagnostic markers are also remained to be further discussed.

In conclusion, we observed that the adoptive transfer of hUCMSCs showed good clinical outcomes combined with other therapeutics for a critically ill COVID-19 patient with severe lung inflammation. Although only 1 case was shown here, it would also be very important to inspire more clinical practice to treat such critically ill COVID-19 patients.

## Author contributions

**Conceptualization:** Min Hu, Chuanyun Qian, Yanfeng Gao

**Data curation:** Mingxi Yang, Panrong Nie, Bing Liang, Haiying Wu

**Formal analysis:** Bing Liang, Tao Li, Junhui Chen

**Funding acquisition:** Min Hu, Chuanyun Qian, Junhui Chen

**Investigation:** Bing Liang, Haiying Wu

**Methodology:** Wenjie Yang^,^ Yanjiao Li, Jianchun Li

**Project administration:** Min Hu, Junhui Chen

**Resources:** Min Hu, Chuanyun Qian

**Supervision:** Junhui Chen, Haiying Wu

**Visualization:** Congtao Yu, Fangang Nie, Zhaoxia Ma

**Writing – original draft:** Chuanyun Qian, Yanfeng Gao, Junhui Chen

**Writing – review & editing:** Min Hu, Yanfeng Gao
